# Using a Bayesian analytic approach to identify county-level ecological factors associated with survival among individuals with early-onset colorectal cancer

**DOI:** 10.1371/journal.pone.0311540

**Published:** 2024-10-29

**Authors:** Sunny Siddique, Laura V. M. Baum, Nicole C. Deziel, Jill R. Kelly, Joshua L. Warren, Xiaomei Ma

**Affiliations:** 1 Department of Chronic Disease Epidemiology, Yale School of Public Health, New Haven, Connecticut, United States of America; 2 Division of Medical Oncology, Department of Medicine, Yale School of Medicine, New Haven, CT, United States of America; 3 Department of Environmental Health Sciences, Yale School of Public Health, New Haven, Connecticut, United States of America; 4 Department of Biostatistics, Yale School of Public Health, New Haven, Connecticut, United States of America; University of Georgia, UNITED STATES OF AMERICA

## Abstract

**Background:**

In the United States (US), incidence of early age of onset colorectal cancer (EOCRC, diagnosed <50 years of age) has been increasing. Using a Bayesian analytic approach, we evaluated the association between county-level ecological factors and survival among individuals with EOCRC and identified hotspot and coldspot counties with unexplained low and high survival, respectively.

**Methods:**

Principal component (PC) analysis was used to reduce dimensionality of 36 county-level social, behavioral, and preventive factors from the Centers for Disease Control and Prevention data. Survival information was derived from the Surveillance, Epidemiology, and End Results Program data from January 1, 2000 to December 31, 2019. The association between the identified PCs and survival was evaluated using multivariable spatial generalized linear mixed models. Counties with residual low and high survival (i.e., unexplained by the PCs) were classified as hotspots and coldspots, respectively.

**Results:**

Four PCs were used to explain the spatial variability in 5-year survival among 75,215 individuals with EOCRC: PC1) poverty, chronic disease, health risk behaviors (β = -0.03, 95% credible interval (CrI): -0.04, -0.03); PC2) younger age, chronic disease-free, minority status (β = -0.01, 95% CrI: -0.02, 0.00); PC3) urban environment, preventive services (β = 0.02, 95% CrI: 0.00, 0.03); and PC4) older age (-0.04, 95% CrI: -0.06, -0.02). Among individuals with distant malignancies, the residual spatial variability remained high for two US counties: 1) Salt Lake County, UT residents experiencing 26.5% (95% CrI: 1.5%, 47.8%) lower odds of survival [hotspot], and 2) Riverside County, CA residents experiencing 37% (95% CrI: 7.97%, 78.8%) higher odds survival [coldspot] after adjustment for county-level factors.

**Conclusions:**

County-level ecological factors are strongly associated with survival among individuals with EOCRC. Yet there is some evidence of survival disparities among individuals with distant malignancies that remain unexplained by the included factors.

## Introduction

In the United States (US), incidence of early age of onset colorectal cancer (EOCRC), defined as colorectal cancer (CRC) diagnosed among individuals <50 years of age, has increased approximately 2% per year since 2011 [1]. Compared to later-onset CRC, EOCRC is characterized by distinct genetic, molecular, and clinical characteristics and survival trajectories. For example, early-onset tumors show a higher prevalence of microsatellite instability, a lower prevalence of *BRAF* mutations, and more often present in women [2]. EOCRC patients are more likely to present with hematochezia and abdominal pain, in part because of the predominance of left-sided tumors [3, 4]. They are more often diagnosed with advanced disease and experience longer time to diagnosis [3]. Compared to older individuals, individuals with EOCRC receive more aggressive treatment, including surgery, multiagent chemotherapy, and adjuvant or neoadjuvant chemotherapy and/or radiotherapy [5–9]. However, those with EOCRC also experience a survival benefit upon adjusting for stage at diagnosis, compared to those diagnosed with CRC at later ages [10].

In CRC broadly, disparities in survival are observed based on race, ethnicity, sex, and geography [1]. However, due to the distinct etiology, clinical characteristics, and survival trajectories of individuals with EOCRC, it is important to assess their survival disparities separately. Previously, a small number of studies have examined EOCRC survival disparities and reported that non-Hispanic (NH) Black men and individuals living in Southern states experience the lowest survival [11–14]. A limited set of county-level factors have been assessed, and prevalence of smoking was found to be a strong predictor of disparities in survival [11]. Factors such as county-level prevalence of smoking may be highly correlated with previously unexplored social and structural factors (i.e., housing, health care, and screening access) with complex implications on survival among individuals with EOCRC. Furthermore, county-level social and structural factors are often spatially correlated due to the tendency of geographically proximal units to share similar characteristics [15]. These social and structural predictors of survival and the spatial correlation among them have been unaccounted for in prior studies, potentially impacting the findings (e.g., confidence intervals may be too narrow).

In this study, we evaluated the association between county-level social, behavioral, and preventive factors and survival among individuals with EOCRC, stratified by stage at diagnosis. We also identified hotspot and coldspot counties where the survival disparities remained unexplained by the county-level factors that were assessed. By including a comprehensive set of county-level social, behavioral, and preventive factors in our analysis and by utilizing a hierarchical Bayesian spatial analytic approach, we accounted for correlation in spatial data that was unaccounted for in prior studies.

## Methods

### Study population

The study population included all individuals who were diagnosed with colorectal cancer (ICD-O-3 Sties: C180-189, C260, C199, C209) under 50 years of age between January 1, 2000 and December 31, 2019 in the 17 population-based registries covered by the National Cancer Institute’s Surveillance, Epidemiology, and End Results (SEER) Program (November 2021 submission) [16]. SEER covers nearly one-third of the US population and includes data on the number of individuals with EOCRC who were alive 5 years after diagnosis in each county [16, 17]. Since EOCRC mortality is reported to have increased approximately 1% per year since the mid-2000s, we chose a study period from the year 2000 to 2019 to capture this shift in the epidemiology of EOCRC [18]. We included only the first primary cancer and removed recurrent malignancies (n = 2,102). Residents of Alaska, Hawaii, and other island counties (n = 220) were excluded because our spatial modeling required all counties to have non-missing covariates and at least one “neighbor” (i.e., bordering county). Remote counties in Alaska had missing covariates, while Hawaii and other islands had no bordering counties.

### Predictors of survival

To identify county-level predictors of survival, aggregated 5-year survival data from SEER were linked with two publicly available datasets using county FIPS codes: 1) 2020 Social Vulnerability Index (SVI) data from the Centers for Disease Control and Prevention’s (CDC) Agency for Toxic Substances and Disease Registry (ATSDR), 2) CDC’s 2020 PLACES data [19–21]. The CDC/ATSDR SVI database and mapping tool was created to assist state, local, and tribal disaster management officials in identifying the locations of their most socially vulnerable populations [19, 22]. Previously utilized in disparity focused studies assessing CRC outcomes, the CDC/ATSDR SVI database is comprised of 16 census variables, representing four domains: socioeconomic status, household composition, racial and ethnic minority status, and housing and transportation [23, 24]. The CDC’s PLACES database uses small area estimation methods to obtain 29 chronic disease measures for the entire US at the county level and has been utilized to assess CRC prevention [25, 26]. Drawing upon Behavioral Risk Factor Surveillance System’s (BRFSS) annual telephone surveys, the PLACES database also includes factors related to health outcomes, prevention, health risk behaviors, and health status [20]. Factors such as prevalence of older individuals, as well as chronic conditions and preventive initiatives among older individuals, were included in our study because they correlate with cancer survival and serve as markers of the social and built environment in which young individuals reside [27–29]. For example, in a county with a high prevalence of older adults receiving CRC screening, it may be challenging to allocate resources to screen younger individuals.

To address the collinearity in the 36 predictor variables that were ascertained from the CDC/ATSDR SVI and PLACES databases (S1 Table), we conducted principal component analysis (PCA). PCA reduces dimensionality in large datasets and allows us to construct a smaller number of variables, called principal components (PCs) that are linear combinations or mixtures of the original variables [30]. Scree plots were used to evaluate the number of PCs to be included in the final model. The optimal number of PCs were chosen based on this plot, interpretability, and Kaiser’s rule (i.e., dropping the components with eigenvalues <1) [31].

### Statistical modeling

Upon identification, PCs were included as predictors in a series of multivariable spatial generalized linear mixed models for areal unit data, with inference carried out in a Bayesian setting using a Markov Chain Monte Carlo sampling algorithm (S1 Appendix) [32, 33]. The response variable was the number of individuals with EOCRC in a county who were alive 5 years after diagnosis, accounting for the total number of individuals diagnosed with EOCRC in the county. This modeling approach ensures that counties with higher number of individuals with EOCRC are weighed more heavily but also improves the estimation of survival in counties with smaller number of cancer cases by taking into consideration the disease burden of neighboring counties. Convergence of the model was assessed using trace plots for individual parameters and analyzing the potential scale reduction factor results, along with the Geweke diagnostic (S1 Appendix) [34, 35].

### Hotspot and Coldspot identification

We extracted posterior samples from the spatial random effect parameters and calculated the posterior means and 95% quantile-based equal-tailed credible intervals (CrI) as summaries for each county. These random effects represent the residual (i.e., after adjusting for the PCs) risk of the outcome and can be useful in understanding spatial patterns that have not been accounted for in the model and/or which may be currently unknown. Counties with negative random effect values (upper 95% CrIs < 0) were considered hotspots, which had generally lower survival among individuals with EOCRC that could not be explained by the PCs. Inversely, counties with positive random effect values (lower 95% CrIs > 0) were considered coldspots (i.e., areas that had generally higher survival among individuals with EOCRC that could not be explained by the PCs) [36, 37].

All spatial models were conducted using the CARBayes package in R Statistical Software (v4.2.4; R Core Team 2022) [32, 38]. Data used in this study were accessed on July 15, 2024. This study was deemed non-human subjects research by the Institutional Review Board of Yale University (Protocol ID: 2000035862) because the data used are publicly available and non-identifiable. Therefore, informed consent was not required.

## Results

We evaluated the association between 36 social, behavioral, and preventive factors and survival among individuals with EOCRC (Table 1). These 36 factors clustered into four PCs, explaining 67.7% of the variance in the original data. Interpretation of the resulting factors was determined based on variables with factor loadings ≥ |0.20|. Trace plots, scale reduction factors, and the Geweke diagnostics showed no obvious signs that the model failed to converge.

**Table 1:** Rotated factor patterns for first four factors from principal component analysis of county-level prevalence of social, behavioral, chronic disease, and preventive factors.

	Principal Component			
	1	2	3	4
	Poverty, chronic disease, health risk behaviors	Young, chronic disease-free, minority status	Urban environment and preventive services	Older age
Eigenvalue	14.48	5.14	2.86	1.87
Percent Variance Explained	40.20%	14.30%	8.00%	5.19%
Cumulative Variance	40.20%	54.50%	62.50%	67.70%
*Regression Coefficient (95% Crl)*				
Overall	-0.03 (-0.04, -0.03)	-0.01 (-0.02, 0.00)	0.02 (0.00, 0.03)	-0.04 (-0.06, -0.02)
In-Situ	-0.15 (-0.23, -0.06)	0.06 (-0.04, 0.15)	0.01 (-0.17, 0.17)	-0.16 (-0.36, 0.04)
Localized	-0.07 (-0.09, -0.05)	0.01 (-0.02, 0.03)	0.04 (-0.01, 0.09)	-0.05 (-0.11, -0.01)
Regional	-0.05 (-0.06, -0.04)	-0.01 (-0.03, 0.00)	0.01 (-0.04, 0.02)	-0.02 (-0.05, 0.01)
Distant	-0.04 (-0.06, -0.03)	0.01 (-0.02, 0.03)	0.01 (-0.04, 0.04)	-0.01 (-0.05, 0.04)
*Sociodemographic Factors*				
% Below 150% of the poverty line	**0.24**	0.01	0.03	0.15
% Unemployment	0.16	0.03	0.17	0.14
% Housing burdened	0.04	0.13	**0.36**	**0.27**
% No HS diploma	**0.21**	0.12	-0.12	0.00
% Uninsured	0.13	0.18	-0.18	-0.03
% >65 years of age	-0.03	**-0.22**	**-0.20**	**0.38**
% <17 years of age	0.04	**0.21**	-0.09	**-0.47**
% Disabled	0.16	**-0.21**	-0.09	**0.21**
% Single parent households	0.14	0.17	**0.22**	-0.15
% with limited English	0.03	**0.34**	-0.08	0.04
% Minority	0.12	**0.34**	0.11	0.04
% Multiunit homes	-0.09	0.18	**0.36**	0.10
% Mobile homes	0.17	-0.07	-0.18	0.08
% Living in crowded housing	0.09	**0.28**	-0.07	0.04
% with no vehicle	0.11	0.05	**0.30**	0.19
% Living in group quarters	0.04	0.02	0.07	**0.22**
*Chronic Diseases*				
% Arthritis	0.19	**-0.24**	0.11	-0.11
% Cancer	-0.10	**-0.35**	0.03	-0.07
% Kidney disease	**0.25**	0.06	0.02	0.07
% COPD	**0.22**	-0.20	-0.01	-0.02
% Coronary heart disease	**0.25**	-0.09	-0.06	0.00
% Asthma	0.16	-0.17	0.18	0.05
% Depression	0.12	**-0.25**	0.01	-0.11
% Diabetes	**0.25**	0.10	0.03	-0.03
% Obesity	**0.20**	-0.06	-0.03	**-0.21**
% Stroke	**0.25**	-0.02	0.06	0.04
*Adverse Health Behaviors*				
% Binge drinking	-0.16	-0.02	-0.17	0.12
% Smoking	**0.21**	**-0.20**	-0.06	-0.04
% With no leisure time physical activity	**0.24**	0.00	-0.04	-0.10
% Not sleeping 7 hours or more	**0.21**	-0.07	0.14	-0.09
*Preventative Services*				
% Aged ≥ 18 years ranking their health poorly	**0.26**	0.03	-0.02	-0.01
% Men aged ≥ 65 years receiving preventive services	-0.15	-0.11	0.20	-0.19
% Women aged ≥ 65 years receiving preventive services	-0.16	-0.11	**0.20**	-0.17
% Aged ≥ 18 receiving routine checkup	0.11	-0.12	**0.34**	**-0.23**
% Ever had colonoscopy (ages 50–75)	-0.15	-0.08	**0.27**	-0.11
% Using fecal occult blood test (ages 50–75)	-0.06	-0.06	0.19	**0.31**

**Boldface** indicates factor loadings ≥ |0.20|

^a^ 95% CrI = 95% Credible Interval

### Patterns in county-level attributes

The four PCs were dominated by: 1) PC1: poverty, chronic disease, and health risk behaviors; 2) PC2: younger age, chronic disease-free, and minority status; 3) PC3: urban environment and preventive services; and 4) PC4: older age (Table 1).

#### PC1: Poverty, chronic disease, health risk behaviors

PC1 included county-level prevalence of extreme poverty, lack of high school education, as well as prevalence of several chronic conditions, including obesity, coronary heart disease, stroke, and diabetes (factor loadings ≥ 0.20). Additionally, prevalence of several health risk behaviors such as smoking, lack of leisure time physical activity and lack of sleeping 7 hours or more loaded heavily on this PC. Furthermore, prevalence of the adult population ranking their health poorly loaded heavily on this PC (factor loading = 0.26). Overall, this PC, containing markers of poverty, chronic disease, and health risk behaviors, showed significant negative association with survival (β = -0.03, 95% CrI: -0.04, -0.03). This PC was also negatively associated with 5-year survival when stratified by stage at diagnosis (Table 1).

#### PC2: Young, chronic disease-free, minority status

On PC2, county-level prevalence of individuals <17 years of age, limited English language proficiency, and minority status loaded positively (factor loadings ≥ 0.20). Prevalence of individuals >65 years of age, disability, having a history of arthritis, cancer, depression, other chronic diseases, and smoking, loaded negatively on this PC (factor loadings ≤ -0.20). This PC was not significantly associated with EOCRC survival (β = -0.01, 95% CrI: -0.02, 0.00).

#### PC3: Urban environment and preventive services

PC3 included factors related to urban living conditions and prevalence of preventive services utilized by individuals living in the county. Factors indicative of urban living conditions included: prevalence of multiunit homes, households that spend 30% or more of annual income on housing costs, and households with no vehicle. Furthermore, factors indicative of preventive services included: prevalence of the population receiving routine primary care checkups, prevalence of the eligible population receiving colonoscopy and fecal occult blood test or fecal immunochemical tests, and men and women up to date with preventive services such as flu vaccine, pneumococcal polysaccharide vaccine, and mammograms. These factors all loaded heavily on PC3 (factor loadings ≥ 0.20), and this PC had significant positive association with survival in both crude (β = 0.02, 95% CrI: 0.00, 0.03) and stage stratified analysis (Table 1).

#### PC4: Older age

Finally, PC4 included prevalence of factors indicative of a high prevalence of older adults: prevalence of individuals > 65 years of age, having disability, and living in group quarters. These factors showed significant negative association with 5-year survival (β = -0.04, 95% CrI: -0.06, -0.02). This PC was also negatively associated with survival upon stratifying by stage, though the association was not statistically significant (except for those with localized disease, β = -0.05, 95% CrI: -0.11, -0.01) (Table 1).

Overall, PC2 and PC4 contained indicators of positive and negative health outcomes, respectively. For example, high prevalence of population <17 years of age loaded positively on PC2 (factor loading = 0.21) and negatively on PC4 (factor loading = -0.47). Furthermore, PC2 contained low prevalence of chronic diseases such as cancer and arthritis (factor loadings < -0.20), whereas PC4 included factors indicative of an aging population, such as high prevalence of population aged 65 years or older and having a disability (factor loadings > 0.20).

### Survival disparities

A total of 75,215 individuals were diagnosed with EOCRC between January 1, 2000 and December 31, 2019 in the following SEER covered states: California, Connecticut, Georgia, Iowa, Kentucky, Louisiana, New Jersey, New Mexico, Utah, and Washington. Among the 609 contiguous counties included in our analysis, county-level ecological factors explained some of the spatial variability in 5-year survival among individuals with in-situ, localized, and regional disease across all included counties. Among individuals with distant disease, we identified two counties where residual spatial variability remained high. Individuals with distant malignancies residing in Riverside County, CA [coldspot] had 37% higher odds of survival (95% CrI: 7.97%, 78.8%) after adjustment for county-level factors. On the other hand, individuals with distant malignancies residing in Salt Lake County, UT [hotspot] experienced 26.5% (95% CrI: 1.5%, 47.8%) lower odds of 5-year survival after adjustment for county-level factors.

## Discussion

This study evaluated the association between county-level social, behavioral, and preventive factors and 5-year survival among individuals with EOCRC and identified 4 PCs that are associated with survival in all included US counties. Furthermore, evidence of unexplained survival disparities was observed among individuals with distant malignancies residing in two US counties. Analysis of these county-level factors is important in understanding how ecological factors contribute to potential survival disparities, elucidating regional patterns not captured by individual-level data, and informing state and national policy geared to reduce disparities and improve health for all.

Previously, disparity-focused studies have observed that individuals with EOCRC residing in Southern and Midwestern states, and rural counties, including the lower Mississippi delta and west central Appalachia, experience low survival following their diagnosis [11, 39–43]. Much of this disparity has been attributable to well established factors. For example, one study identified prevalence of diabetes as the most important risk factor in predicting EOCRC outcomes [12]. Other studies have established prevalence of smoking as a strong contributor to EOCRC survival disparities [11, 43]. Findings from our study are novel, because we accounted for these factors and additionally identified a potential hotspot in the state of Utah and coldspot in the state of California. Survival patterns among individuals with distant malignancies residing in these two counties remain largely unexplained by our included ecological factors. Notably, Salt Lake County is the most populous county in Utah and Riverside County is the fourth most populous county in California. Therefore, future research is warranted to elucidate the individual-level clinical, demographic, and socioeconomic factors as well as the diverse healthcare landscapes in these counties that may be contributing to the survival disparities observed.

The PCs identified in this study showed strong correlations among county-level social, behavioral, and preventive factors whose disproportionate burden may drive survival disparities–offering a window into ways in which novel interventions may be developed to target complex, multidimensional risk factors. The first PC, accounting for over 40% of variance, included poverty, chronic diseases, and health risk behaviors and revealed several modifiable factors that may be targeted by health promotion initiatives. For example, lack of leisure time physical activity, lack of sleeping 7 hours or more, and smoking loaded heavily on this PC, along with prevalence of several chronic diseases, including diabetes and coronary heart disease. Interventions to reduce one or more of these risk behaviors and management of chronic conditions may ultimately improve clinical outcomes of individuals with EOCRC and improve survival.

This study has several limitations. To protect individual identity and privacy, geographic information from SEER was aggregated and made publicly available at the county level. Therefore, the survival disparities that we observed may be attributable to individual level factors, such as proximity to cancer care and insurance status [44]. Although several prior studies have established county-level factors as adequate measures to identify geographic disparities, our study may be prone to the modifiable areal unit problem–a statistical biasing effect that occurs when observed associations between explanatory factors and outcomes vary based on the scale of the chosen geographic unit [11, 12, 45, 46]. Therefore, conducting this analysis using smaller geographic units, such as census tracts, may reveal further insights into specific areas within counties that are experiencing survival disparities. Next, as with all self-reported sample surveys, the covariate measures utilized in this study might be subject to systematic error resulting from noncoverage, nonresponse, or measurement bias due to small population size of counties. The CDC uses a multilevel regression and poststratification (MRP) approach that links geocoded health surveys and high spatial resolution demographic and socioeconomic data. CDC’s internal and external validation studies confirm the strong consistency in the MRP approach to estimating county-level prevalence of the covariates utilized in this study [19–21]. Furthermore, the potential for residential mobility has not been considered in this study. During the time period included in this study (from January 1, 2000 to December 31, 2019), individuals with EOCRC may have changed counties and their residential environment may have improved or declined [47–49]. Changing the residential environment may have a separate effect on health outcomes, possibly due to negative factors, such as stress related to moving and disruption of established social connections or behavioral routines, and positive factors such as moving to a more walkable neighborhood [49, 50]. Lastly, since our study covered nearly two decades of data, changes in the overall healthcare landscape that may have impacted healthcare access among individuals with EOCRC, such as the implementation of the Affordable Care Act, are not accounted for in our study.

## Conclusion

In the majority of SEER covered counties in the US, county-level social, behavioral, and preventive factors are associated with 5-year survival among individuals with EOCRC and help explain some of the spatial variability in survival, after accounting for stage at diagnosis. Yet in two US counties, the residual spatial variability remained high among individuals with distant EOCRC, likely due to their large heterogenous populations and differences in individual level clinical, demographic, and socioeconomic characteristics. The rising burden of EOCRC warrants the incorporation of ecological data in future studies, as ecological factors encapsulate a person’s multiple interactions with the social and physical environment that can result in lasting health impact. Results from this study may be utilized to help identify geographic areas with lower than expected survival among individuals with EOCRC, and design community-level interventions to address survival disparities. A similar approach may also be applicable to the study of other chronic diseases.

## Supporting information

S1 Appendix Supplementary methods. Descriptions of spatial modeling approach.
(DOCX)

S1 Table Detailed descriptions of data source and covariates used to identify hotspots and coldspots. (DOCX)
