# Supplementary material for: Using a Bayesian analytic approach to identify county-level ecological factors associated with survival among individuals with early-onset colorectal cancer
Source: PLoS One. 2024 Oct 29;19(10):e0311540. doi: 10.1371/journal.pone.0311540 (PMC11521299; doi:10.1371/journal.pone.0311540)
Supplement: S1 Appendix — Descriptions of spatial modeling approach. (DOCX) [file pone.0311540.s001.docx]

**S1 Appendix: Supplementary Methods – Descriptions of spatial modeling approach**

Markov Chain Monte Carlo Sampling

Using Bayesian statistics allows us to more easily fit hierarchical models, like the spatial model proposed in this work. To fit these types of models, we rely on computational approaches. Specifically, Markov chain Monte Carlo (MCMC) sampling methods allow us to fit the model to the data and learn about the model parameters using an iterative sampling algorithm. These approaches yield samples from posterior distributions of the model parameters which are then used to estimate the parameters and their uncertainty. A posterior distribution combines the observed data and the prior distributions using Bayes’ theorem, which in this case were selected to be weakly informative so that the inference was driven by the data rather than prior beliefs. All of the inference displayed in the manuscript represents summaries of posterior samples for the different model parameters.

Multivariable spatial generalized linear mixed models for areal unit data

In our multivariable Bayesian models, we included the chosen number of principal components (PCs) as predictors and the number of individuals with early-onset colorectal cancer (EOCRC) alive 5 years after diagnosis as the outcome variable, accounting for the total number of individuals diagnosed with EOCRC in the county to ensure that counties with higher number of individuals with EOCRC contribute more information to model estimation. The outcome variable was derived by the Surveillance, Epidemiology, and End Results Program through survival analysis. Spatial autocorrelation in the outcome was modelled using a set of random effect parameters that are assigned a conditional autoregressive prior distribution [1]. This distribution specifies that neighboring counties are potentially more similar *a priori* but allows the data to determine the amount of spatial correlation that is present. We created a neighborhood matrix using the basic binary coding style (i.e., two counties are neighbors if they have touching borders). Since MCMC methods are iterative, it requires us to run the algorithms for many iterations and determine at what point we have reached convergence. In our specific application, 300,000 samples collected from each chain were needed to simultaneously reach convergence and ensure that we collected enough samples post-convergence to accurately estimate the parameters in the model. Furthermore, the first 100,000 samples were subsequently removed as the burn-in period, which are the iterations from the algorithm that are discarded prior to convergence of the model. The remaining 200,000 samples were thinned by 100 to reduce their autocorrelation, resulting in 6,000 samples for inference combined across the three analyses [2]. This autocorrelation refers to the fact that in iterative MCMC algorithms, the collected posterior samples are often correlated across iteration. Since we prefer to have independent samples from the posterior distributions, we thin the collected samples to reduce this correlation. Running these repeated samples is necessary for the models to converge and allows us to ensure robust statistical inference.

Assessment of Model Convergence

We used trace plots, scale reduction factors, and the Geweke diagnostic to assess model convergence [3]. While convergence is difficult to prove formally, these tools are useful for detecting signs of non-convergence. Convergence of the model is required in order to obtain samples from the joint posterior distribution for all parameters needed to make accurate statistical inference. Trace plots visually depict the value of the collected posterior sample (y-axis) against the iteration number (x-axis). A line with no trend, jumps, or gaps is often indicative of model convergence. The potential scale reduction factor is the factor by which between-chain variation might decline under future simulations. Values <1.1 are indicative of model convergence. Finally, the Geweke diagnostic compares the mean of the samples drawn from the end of a chain to the mean of the samples at the beginning of the chain while accounting for autocorrelation when calculating the p-value.

**References**

1. Leroux B, Lei X, Breslow N. Estimation of Disease Rates in Small Areas; A New Mixed Model for Spatial Dependence. Statistical Models in Epidemiology, the Environment and Clinical Trials. New York: Springer-Verlag; 2000. p. 179-91.
2. Lee D. CARBayes: An R Package for Bayesian Spatial Modeling with Conditional Autoregressive Priors. J Stat Softw. 2013;55(13):1-24.
3. van de Schoot R, Kaplan D, Denissen J, Asendorpf JB, Neyer FJ, van Aken MAG. A gentle introduction to bayesian analysis: applications to developmental research. Child Dev. 2014;85(3):842-60.
