# Supplementary material for: Using a Bayesian analytic approach to identify county-level ecological factors associated with survival among individuals with early-onset colorectal cancer
Source: PLoS One. 2024 Oct 29;19(10):e0311540. doi: 10.1371/journal.pone.0311540 (PMC11521299; doi:10.1371/journal.pone.0311540)
Supplement: S1 Table — (DOCX) [file pone.0311540.s002.docx]

| **S1 Table. Detailed descriptions of data source and covariates used to identify hotspots and coldspots** | | |
| --- | --- | --- |
| Variable | Description | Data Source |
| *Sociodemographic Factors* | | |
| % Below 150% of the Poverty Line | Percent of individuals with income below 150 percent of poverty level | CDC/ASTDR SVI |
| % Unemployment | Percent of individuals (age 16+) who are unemployed | CDC/ASTDR SVI |
| % Housing Burdened | Percent of occupied housing units with income less than $75,000 that utilized 30% or more of their income toward housing costs | CDC/ASTDR SVI |
| % No HS Diploma | Percent of individuals (age 25+) with no high school diploma | CDC/ASTDR SVI |
| % Uninsured | Percent of total civilian noninstitutionalized population who are uninsured | CDC/ASTDR SVI |
| % >65 years of age | Percent of individuals aged 65 and older | CDC/ASTDR SVI |
| % <17 years of age | Percent of individuals aged 17 and younger | CDC/ASTDR SVI |
| % Disabled | Percent of civilian noninstitutionalized population with a disability | CDC/ASTDR SVI |
| % Single parent households | Percent single-parent households with children under 18: Male/Female householder, no spouse or partner present, with own children under 18 years | CDC/ASTDR SVI |
| % with Limited English | Percent of individuals (age 5+) who speak English "less than well" | CDC/ASTDR SVI |
| % Minority | Percent of individuals identifying as minority: (Hispanic or Latino (of any race); Black and African American, Not Hispanic or Latino; American Indian and Alaska Native, Not Hispanic or Latino; Asian, Not Hispanic or Latino; Native Hawaiian and Other Pacific Islander, Not Hispanic or Latino; Two or More Races, Not Hispanic or Latino; Other Races, Not Hispanic or Latino) | CDC/ASTDR SVI |
| % Multiunit homes | Percent of individuals housed in structures with 1 or more units | CDC/ASTDR SVI |
| % Mobile Homes | Percent of individuals residing in mobile homes | CDC/ASTDR SVI |
| % Living in crowded housing | Percent of occupied housing units with more people than rooms | CDC/ASTDR SVI |
| % with No Vehicle | Percent of households with no vehicle available | CDC/ASTDR SVI |
| % Living in Group Quarters | Percent of individuals residing in group quarters | CDC/ASTDR SVI |
| *Chronic Diseases* | | |
| % Arthritis | Percent of respondents aged ≥ 18 years who answered “yes” or “no” to the following question: “Have you ever been told by a doctor, nurse, or other health professional that you have some form of arthritis, rheumatoid arthritis, gout, lupus, or fibromyalgia?” | BRFSS via CDC PLACES initiative |
| % Cancer | Percent of respondents aged ≥ 18 years who report ever having been told by a doctor, nurse, or other health professional that they have any other types (besides skin) of cancer. | BRFSS via CDC PLACES initiative |
| % Kidney disease | Respondents aged ≥18 years who report ever having been told by a doctor, nurse, or other health professional that they have kidney disease. | BRFSS via CDC PLACES initiative |
| % COPD | Respondents aged ≥18 years who report ever having been told by a doctor, nurse, or other health professional that they had chronic obstructive pulmonary disease (COPD), emphysema, or chronic bronchitis. | BRFSS via CDC PLACES initiative |
| % Coronary Heart Disease | Respondents aged ≥18 years who report or do not report ever having been told by a doctor, nurse, or other health professional that they had angina or coronary heart disease | BRFSS via CDC PLACES initiative |
| % Asthma | Respondents who answer “yes” both to both of the following questions: “Have you ever been told by a doctor, nurse, or other health professional that you have asthma?” and the question “Do you still have asthma?” | BRFSS via CDC PLACES initiative |
| % Depression | Respondents aged ≥18 years who answered “yes” or “no” to the following question: “Have you ever been told by a doctor, nurse, or other health professional that you had a depressive disorder (including depression, major depression, dysthymia, or minor depression)?” | BRFSS via CDC PLACES initiative |
| % Diabetes | Respondents aged ≥18 years who report ever been told by a doctor, nurse, or other health professional that they have diabetes other than diabetes during pregnancy. | BRFSS via CDC PLACES initiative |
| % Obesity | Respondents aged ≥18 years who have a body mass index (BMI) ≥30.0 kg/m² calculated from self-reported weight and height. | BRFSS via CDC PLACES initiative |
| % Stroke | Respondents aged ≥18 years who report ever having been told by a doctor, nurse, or other health professional that they have had a stroke. | BRFSS via CDC PLACES initiative |
| *Health Risk Behaviors* | | |
| % Binge drinking | Adults aged ≥18 years who report having five or more drinks (men) or four or more drinks (women) on an occasion in the past 30 days. | BRFSS via CDC PLACES initiative |
| % Smoking | Respondents aged ≥18 years who report having smoked ≥100 cigarettes in their lifetime and currently smoke every day or some days. | BRFSS via CDC PLACES initiative |
| % with no leisure time physical activity | Respondents who answered “no” to the following question: “During the past month, other than your regular job, did you participate in any physical activities or exercises such as running, calisthenics, golf, gardening, or walking for exercise?” | BRFSS via CDC PLACES initiative |
| % Not sleeping 7 hours or more | Respondents aged ≥18 years who report usually getting insufficient sleep (<7 hours for those aged ≥18 years, on average, during a 24-hour period) | BRFSS via CDC PLACES initiative |
| *Preventive Services* | | |
| % Ranking their health poorly | Respondents aged ≥ 18 years who report their general health status as “excellent,” “very good,” “good,” “fair,” or “poor” (excluding unknowns and refusals). | BRFSS via CDC PLACES initiative |
| % Men receiving preventative services | Percent of men men aged ≥65 years reporting having received all of the following: an influenza vaccination in the past year; a pneumococcal vaccination (PPV) ever; and either a fecal occult blood test (FOBT/FIT) within the previous year, a FIT-DNA test within the previous 3 years, a sigmoidoscopy within the previous 5 years, a sigmoidoscopy within the previous 10 years with a FOBT in the previous year, a colonoscopy within the previous 10 years, or a CT colonography (virtual colonoscopy) within the previous 5 years. | BRFSS via CDC PLACES initiative |
| % Women receiving preventative services | Percent of women aged ≥65 years reporting having received all of the following: an influenza vaccination in the past year; a pneumococcal vaccination (PPV) ever; either a fecal occult blood test (FOBT/FIT) within the previous year, a FIT-DNA test within the previous 3 years, a sigmoidoscopy within the previous 5 years, a sigmoidoscopy within the previous 10 years with a FOBT in the previous year, a colonoscopy within the previous 10 years, or a CT colonography (virtual colonoscopy) within the previous 5 years; and a mammogram in the past 2 years. | BRFSS via CDC PLACES initiative |
| % Receiving routine checkup | Respondents aged ≥18 years who report having been to a doctor for a routine checkup (e.g., a general physical exam, not an exam for a specific injury, illness, condition) in the previous year. | BRFSS via CDC PLACES initiative |
| % Ever had colorectal endoscopy | Respondents aged 50-75 years who report having a colonoscopy within the previous 10 years; a sigmoidoscopy within the previous 5 years; or a CT colonography within previous 5 years. | BRFSS via CDC PLACES initiative |
| % Using fecal occult blood test | Respondents aged 50-75 years who report having had a fecal occult blood test (FOBT/FIT) within the previous year; or a FIT-DNA test within the previous 3 years. | BRFSS via CDC PLACES initiative |

Abbreviations:

CDC: Centers for Disease Control and Prevention

ATSDR/SVI: Agency for Toxic Substances and Disease Registry / Social Vulnerability Index

BRFSS: Behavioral Risk Factor Surveillance System
